# MR Molecular Image Guided Treatment of Pancreatic Cancer with Targeted ECO/miR-200c Nanoparticles in Immunocompetent Mouse Tumor Models

**DOI:** 10.1007/s11095-024-03762-7

**Published:** 2024-08-28

**Authors:** Victoria Laney, Ryan Hall, Xueer Yuan, Emma Hampson, Augusta Halle, Grace Yeung, Kristen-Weber Bonk, Suneel Apte, Jordan Winter, Ruth Keri, Zheng-Rong Lu

**Affiliations:** 1 Department of Biomedical Engineering, Case Western Reserve University, Wickenden 427, 10900 Euclid Avenue, Cleveland, OH 44106 USA; 2 Lerner Research Institute, Cleveland Clinic, Cleveland, OH 44106 USA; 3 Surgical Oncology, The University Hospitals of Cleveland, Cleveland, OH 44106 USA; 4 Case Comprehensive Cancer Center, Case Western Reserve University, Cleveland, OH 44106 USA

**Keywords:** ECO, magnetic resonance molecular imaging, miR-200c, MT218, pancreatic cancer

## Abstract

**Objective:**

Pancreatic ductal adenocarcinoma (PDAC) is characterized by desmoplasia due to increased deposition of extracellular matrix (ECM) proteins. This work investigates the efficacy of targeted ECO/miR-200c nanoparticles (ELNP) on ECM remodeling in PDAC and tumor proliferation with MR molecular imaging (MRMI) with MT218 in immunocompetent mouse models.

**Methods:**

The miR-200c mediated regulation of EMT markers was measured in PDAC cells *in vitro*. Wild-type mice bearing mutated KRAS-driven KPC subcutaneous or orthotopic tumors were dosed weekly with RGD-ELNP/miR-200c at 1 mg-RNA/kg for a total of 4 doses. We utilized MT218-MRMI to non-invasively monitor the alteration of tumor ECM EDN-FN levels by miR-200c and tumor response to the treatment. The changes were also validated by posthumous histopathology.

**Results:**

Transfection of PDAC cells with ELNP/miR-200c downregulated the expression of FN1 and EDB-FN and some mesenchymal markers, inhibiting 3D spheroid formation and migration of KPC PDAC cells. RGD-ELNP/miR-200c treatment resulted in significant signal reduction in the MT218 enhanced MRMI images of both subcutaneous and orthotopic KPC tumors compared to those prior to treatment and treated with a non-specific control. MT218-MRMI results were suggestive of EDB-FN downregulation in tumors, which was later confirmed by immunohistochemistry. Tumor growth in subcutaneous tumors was significantly attenuated with RGD-ELNP/miR-200c and was an observed trend in orthotopic tumors. Substantial necrosis and remodeling were observed in both models treated with RGD-ELNP/miR-200c based on H&E staining.

**Conclusion:**

These results demonstrate the feasibility of RGD-ELNP/miR-200c in modulating PDAC ECM and restraining tumor growth and the utility of MT218-MRMI for non-invasively monitoring miR-200c efficacy.

**Supplementary Information:**

The online version contains supplementary material available at 10.1007/s11095-024-03762-7.

## Introduction

Pancreatic ductal adenocarcinoma (PDAC) accounts for more than 90% pancreatic cancer malignancies, and is the fourth leading cause of cancer related deaths worldwide [1, 2]. The 5-year survival rate for PDAC is a mere 11.5%, and incidence of PDAC is projected to double over the next decade [3–5]. Outcomes for PDAC treatment are largely determined by the histological stage at the time of diagnosis, which significantly impacts clinical decision-making [6, 7]. However, most patients are diagnosed when the disease has locally invaded or metastasized [8–11]. The only curative therapy for PDAC is surgery followed by neoadjuvant therapy, but less than 20% of patients are eligible for surgical resection and 60–80% of patients experience reoccurrence in the months after surgical intervention [12, 13]. Notably, PDAC is also resistant to traditional chemotherapy and radiotherapy monotherapy regimens [5, 14, 15]. Densely packed pancreatic cancer cells, cell stemness, and desmoplasia in the tumor microenvironment play key roles in the chemoresistance and overall aggressiveness of PDAC [14, 16]. Immunosuppression and heterogeneity within tumors further exacerbate therapeutic strategies to mitigate tumor invasion through the ductal tissue and metastases into other organs. Thus, there is a critical need for more efficacious therapies to effectively treat PDAC.

The invasive and desmoplastic characteristics of PDAC have been linked to oncogenic pathway, epithelial-mesenchymal transition (EMT) [17–19]. EMT is a reversible biological process whereby tumor cells undergo a phenotypic change from the less invasive epithelial state to an invasive mesenchymal state. EMT is regulated by a complex network of proteins and cells influenced by epigenetic modifications and transcriptional control [17]. Transcription regulators such as microRNAs, including the miR-200 family, have been identified as epithelial markers and suppressors of EMT [20, 21]. miR-200c, a member of miR-200 family, has been shown to reduce tumor growth in various cancers, including breast cancer and osteosarcoma [22, 23]. In PDAC specifically, low miR-200c expression is also associated with EMT and metastatic phenotype, but therapeutic strategies aiming to upregulate or deliver miR-200c as a tumor suppressor have yet to be thoroughly explored [24–26]. Since miR-200c regulates multiple oncotargets, including zinc finger E-box binding homeobox 1 (ZEB1), fibronectin 1 (FN), and extradomain B fibronectin (EDB-FN), delivery of miR-200c to pancreatic malignancies could regulate multiple tumorigenic pathways involved in PDAC progression and eventually promote a less aggressive phenotype. Since miR-200c has more of a tuning effect to existing pathways, we anticipate that increased miR-200c delivery would downregulate oncotargets that are upregulated during tumorigenesis and EMT.

While miR-200c delivery holds promise for treating PDAC more effectively than conventional therapies, miR-200c is a double-stranded RNA and is therefore prone to rapid degradation when delivered in its naked form. Utilizing environmentally responsive nanocarriers could provide a more effective method for delivering miR-200c. Previously, we established multifunctional pH-sensitive amphiphilic lipid, ECO, could serve as a versatile and effective platform for the delivery of therapeutic nucleic acids such as microRNA [23, 27, 28]. The use of ECO lipid nanoparticle (ELNP) formulations addresses challenges associated with poor stability of certain nucleic acid sequences, as demonstrated by the incorporation of an unmodified miR-200c duplex [23]. To improve therapeutic delivery and payload release we targeted α_v_β_3_ integrins, which are upregulated in the angiogenic PDAC environment [29]. Moreover, we have established that ELNP decorated with the integrin targeting cyclic RGD peptide facilitates efficient delivery of miR-200c cargo for triple-negative breast cancer (TNBC) therapy [23, 27]. RGD as a targeting peptide can also be leveraged for PDAC, since it targets integrins overexpressed on pancreatic epithelial cells, and has also been shown to be upregulated during angiogenesis [30, 31]. Thus, ELNP offers a biocompatible lipid-based system that can efficiently and systemically deliver miR-200c to treat PDAC.

A distinguishing characteristic of PDAC is its highly fibrotic nature, marked by desmoplasia, where the tumor microenvironment (TME) produces a dense extracellular matrix (ECM) filled with active fibroblasts and overexpressed fibrotic proteins such as collagen and FN [32]. This desmoplastic environment acts as a physical and metabolic barrier to therapy, promotes heterogeneity across the tumor and promotes immune suppression [16, 33]. FN is an abundant extracellular matrix protein, and its overexpression in cancer is often associated with tumor growth, migration, invasion, and stiffness [34, 35]. EDB-FN is an alternatively spliced oncofetal isoform of FN involved in neovasculatization, EMT, cell motility, and invasion [35, 36]. EDB-FN is overexpressed in aggressive tumors, including PDAC, with nominal expression in normal adult tissues [27, 28]. The *FN1* gene and thus, FN and EDB-FN proteins, are targets of miR-200c. Successful delivery of miR-200c would produce notable changes in expression of both FN and EDB-FN proteins in the TME [37, 38]. Thus, they can act as molecular markers to non-invasively monitor tumor response to miR-200c therapy. To this end, we developed a novel EDB-FN-targeting contrast agent called ZD2-N3-Gd(HP-DO3A) or MT218 for MR molecular imaging (MRMI) of cancer [27, 30]. Previously, we demonstrated that MT218-MRMI targeting ELNP/miR-200c effectively reduced EDB-FN levels in the ECM of triple-negative breast cancer (TNBC), inhibiting tumor proliferation [21]. Furthermore, using MT218-MRMI allowed for accurate visualization and quantification of EDB-FN levels in TNBC tumors. In this study, we assessed the therapeutic efficacy of systemically delivering miR-200c-3p with RGD-targeted ELNP to modulate the fibrotic TME of PDAC using MT218-MRMI. We focused on changes in EDB-FN levels and evaluated how these stromal changes in the pancreatic ECM affected tumor responses in immunocompetent mouse models of PDAC.

## Methods

### Nanoparticle Formulation

**Scheme 1.**
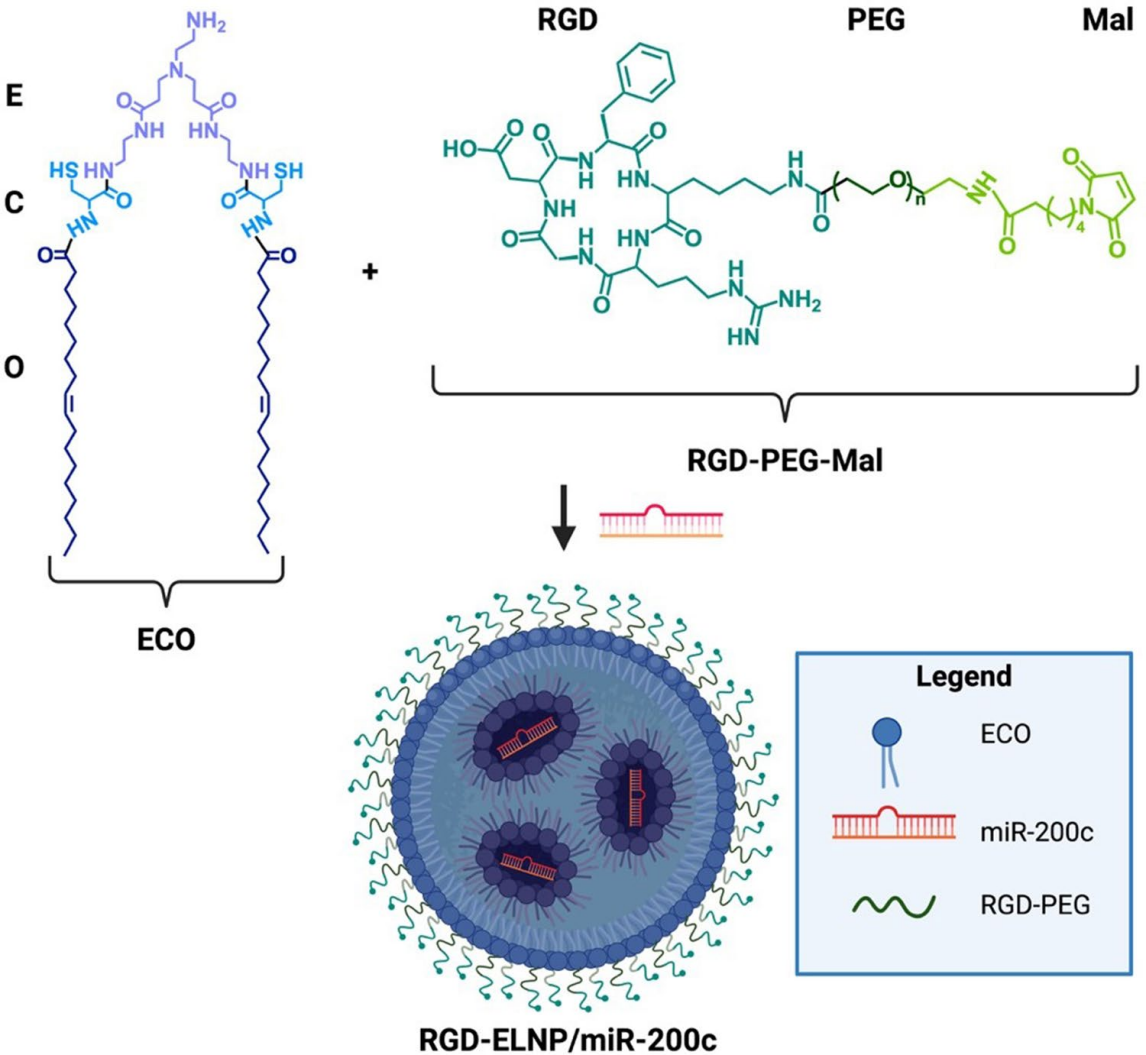
Key chemical structures for RNA nanoparticles. Top shows ECO, ethylene diamine head (E), cysteine residues (C) and oleic acid tails, and RGD-PEG-Mal structures. Bottom of figure shows ELNP nanoparticle of with miR-200c cargo (RGD-ELNP/miR-200c).

ECO and RGD-PEG-Mal were synthesized as previously described by Schilb *et al*. (Scheme 1) [23, 39]. Briefly, the manufacture technique for our self-assembly nanoparticles is as follows: targeted ligands were mixed under agitation with ECO alone, allowing for lipid carrier formation. Then miRNA was added and further mixed for 30 min to allow for self-assembly. Both the miR-200c duplex and non-specific control siRNA (siNS) were purchased form IDT (Newark, NJ). Stock solutions of ECO in ethanol with concentrations of 5 and 50 mM were used to formulate ELNP for *in vitro* and *in vivo* experiments, respectively. To target α_v_β_3_ integrins, cyclic RGD peptide was directly conjugated to NHS-PEG-Mal. RGD-PEG-ECO/miR-200c nanoparticles (RGD-ELNP/miR-200c) were formulated through the self-assembly process of RGD-PEG-MAL, ECO, and an unmodified miR-200c duplex at an N/P ratio of 8, as detailed in previous studies [28, 39, 40]. Non-specific control nanoparticles, RGD-ELNP/siNS were similarly formulated using the non-specific siRNA duplex. Targeted RGD-ELNP nanoparticles were then supplemented with 5% sucrose in preparation for storage at -80 ºC. The size and zeta potential of the ELNP were determined via dynamic light scattering (DLS) and electrophoretic light scattering (ELS) in water using a Litesizer 500 system (Anton Paar USA, Ashland, VA, USA). Particles were measured again for size, charge, and encapsulation after 2 weeks to determine stability.

### Agarose Gel Electrophoresis

Agarose gels were prepared with 1:10 w/v agarose to 0.5 × Tris borate-EDTA buffer (Thermo Fisher Scientific, Waltham, MA). Ethidium bromide was used for visualizing DNA fragments. Samples were mixed with 6 × DNA-loading dye (Thermo Fisher Scientific) prior to loading to allow for visual tracking. The samples were loaded into the gel and run for 20 min at 100 V. The gels were imaged using a ChemiDoc XRS system (Bio-Rad, Hercules, CA).

### Cell Culture and *In Vitro* Transfection

Murine Kras^G12D/+^; P53^R172H/+^; Pdx1-Cre PDAC cells (KPC-K8484 herein KPC) derived from an inducible murine model were generously provided by Dr. Jordan Winter (University Hospitals of Cleveland, OH). Cells were cultured in an incubator at 37°C and 5% CO_2_ with normal RMPI media (RPMI medium supplemented with 10% fetal bovine serum (FBS) and 1% Penicillin/Streptomycin (Thermo Fisher Scientific)). For *in vitro* transfection, KPC cells were cultured in 6-well plates at 1 × 10^6^ cells/well. After being cultured in serum-free RPMI for 6 h, cells were washed and transfected with 25 µM untargeted ELNP/miR-200c or ELNP/siNS in normal RPMI media. Controls wells were washed and then refreshed with normal RPMI media. In a given 6-well plate, each group, control, ELNP/miR-200c and ELNP/siNS was seeded in 2 wells. Cells were harvested, split, and spun down into cell pellets after 24 h for subsequent assays. *In vitro* transfection was conducted in triplicate, where a single 6-well plate was considered a single experiment.

### qRT-PCR

RNA extraction was performed using an RNeasy Plus RNA extraction kit (Qiagen, Germantown, MD) according to the manufacturer’s instructions. Reverse transcription was performed using the SuperScript IV reverse transcriptase kit (Invitrogen, Carlsbad, CA). qRT-PCR was performed using SYBR Green Master Mix (AB Biosciences, Allston, MA, USA) on a Bio-Rad CFX96 qRT-PCR system. mRNA-fold expression was determined with the 2^−ΔΔCt^ method using 18 s as the housekeeping gene. Forward and reverse primers used during this study can be found in Supplemental Table 1 (EDB-FN, FN1, Vimentin, E-cadherin, TGFβ1, ZEB1, B catenin, N-cadherin, and 18 s).

### Western Blotting

Cell pellets were digested in RIPA buffer with protease inhibitors (Roche, Basel, Switzerland). Proteins were then separated by SDS-PAGE and transferred onto a nitrocellulose membrane. Membranes were blocked with 5% bovine serum albumin in Tris-buffered saline containing 0.1% Tween-20 (TBST) for 1 h. Membranes were incubated on ice overnight with primary antibodies to ZEB1, E-cadherin, N-cadherin, vimentin, fibronectin, EDB-FN, GAPDH and β-actin (Absolute Antibody, Oxford, UK). Following primary antibody incubation, membranes were washed with TBST and incubated with secondary anti-rabbit IgG antibody (Cell Signaling Technology, Danvers, MA) at room temperature for 1 h. MagicMark (Thermo Fisher Scientific) and Kaleidoscope (Bio-Rad) were used as protein standards and ladders. Membranes were activated using Signal Fire Plus ECL reagent (Cell Signaling Technologies, Danvers, MA) and imaged using Gel Doc Gel Documentation System (Bio-Rad). Protein band intensities were quantified with ImageLab software (Bio-Rad).

### 3D Spheroids/ECM Protein Staining

Following *in vitro* transfection, 1 × 10^4^ cells from each group were cultured on Matrigel (Corning, Somerville, MA) to facilitate ECM and 3D spheroid formation. To allow for sufficient spheroid growth, cells were incubated for 24 h until spheroids formed. To determine EDB-FN levels and distribution in 3D culture, spheroids were incubated for 20 min with Hoescht 33,342 and ZD2-Cy5.5 to stain for cell nuclei and EDB-FN, respectively. Spheroids were then imaged with fluorescence microscopy using an Olympus FluoView FV1000 confocal microscope (Olympus, Tokyo, Japan). Fluorescence from Cy5.5 and DAPI channels were quantified in FIJI (ImageJ, NIH, Bethesda, MD).

### Functional Assays/Wound Assay

Following transfection, cells were used for wound healing assay. Two intersecting scratches were made in each well using a sterile 10 μL pipette tip. Wound closure was evaluated and imaged immediately after the first scratch and imaged again at timepoints 12 h and 24 h. Acquired images were quantified in FIJI (ImageJ) to assess scratch wound closure.

### Animal Care and Tumor Establishment

C57BL/6 J mice (both sexes, male and female) were used to develop orthotopic and subcutaneous PDAC models. Mice were housed in the animal facility at Case Western Reserve University (CWRU) according to an animal protocol approved by the CWRU Institutional Animal Care and Use Committee (IACUC) and conformed to recommendations of the American Veterinary Medical Association Panel on Euthanasia. Terminal points for mice were a tumor mass of 10% of total body weight, or adverse side effects as outlined in an IACUC-approved protocol. Otherwise, mice were euthanized at day 42 (42 days after implantation) following the final MRMI acquisition.

For subcutaneous tumors, a 1:1 DPBS and Matrigel (18–20 mg/ml) mixture containing 1 × 10^5^ KPC cells was prepared and inoculated into the left flank. Tumors were allowed to grow for 2 weeks until they reached an average size of 100 mm^3^, after which mice were randomized into experimental groups of n = 6 (3 M and 3 F). Tumor volume was calculated as (length x width^2^)/2 based on caliper measurements.

Orthotopic intrapancreatic tumors were established via pancreatic laparotomy under anesthesia. A total of 10 mice were in each group (n = 10/group, 5 M and 5 F). KPC cells (5 × 10^3^) suspended in 2:1 v/v DPBS and Matrigel solution, which was then injected directly into the head of the pancreas. Surgical wounds were sutured and stapled, and mice were monitored daily for 5 days post-surgery. Staples were removed on day 10 prior to MRMI. Intrapancreatic tumor establishment was verified on day 10 by T_1_-weighted axial MRMI with MT218 (Molecular Theranostics, Cleveland, OH). Mice were randomized and kept in the study if tumors were a measurable volume of 25 mm^3^ or greater. Tumor volume was measured in FIJI through region of interest (ROI) area measurement. An ROI was taken for each image slice containing tumor tissue. Tumor volume was calculated by summing individual areas from the ROIs and multiplying by 1.2. The multiplication factor was used to account for the 1 mm slice thickness and the 0.2 mm slice gap from MRMI acquisition. Tumors were identified by visual assessment by trained lab members.

### *In Vivo* Treatment of PDAC with miR-200c

RGD-ELNP/miR-200c and RGD-ELNP/siNS were prepared and characterized as previously described and stored in -80 ºC until dosing [23]. Nanoparticles were stored for no longer than 2 weeks and were recharacterized prior to injection. Nanoparticle injections were conducted weekly at days 14, 21, 28 and 35. RGD-ELNP/RNA (1 mg RNA/kg) were administered i.v. through the tail vein at a volume of 100 µL.

### MRMI and Treatment Monitoring

Magnetic resonance molecular imaging (MRMI) was performed on a 3 T MRS 3000 scanner (MR Solutions, Surrey, UK). T_1_-weighted fast spin echo (FSE, 2D, axial plane, T_R_ = 305 ms, T_E_ = 11 ms, FA = 90°, FOV = 40 × 40 mm, sampling period = 300 ms, slice thickness = 1 mm, slice gap = 0.2 mm, N_av_ = 4, matrix = 256 × 256) and fast low angle shot (3D, coronal plane acquisition, T_R_ = 16 ms, T_E_ = 1 ms, FA = 25°, FOV = 128 mm, sampling period = 250 ms, N_av_ = 2, matrix = 256 × 256) images were taken prior to contrast injection (pre-contrast) as well as at 10, 20, and 30 min post-contrast injection. The contrast agent MT218 was administered via i.v. injection (through a tail vein catheter) at 0.08 mmol/kg. Mice were imaged at 3 timepoints following tumor establishment: day 10, day 26 and day 42. The imaging data was transferred to DICOM format and analyzed in FIJI (ImageJ), Horos and ITK-segment for tumor progression and signal changes through contrast-to-noise [$CNR= \frac{{s}_{tumor}-{s}_{muscle}}{{\sigma }_{noise}}]$ as well as tumor size and volume measurements. ROI for tumor, muscle, and noise in the T_1_-weighted axial FSE were manually determined by at least two independent reviewers who were blinded to the treatment groups.

### Histopathology

Histological slides were prepared by formalin fixation or snap-freezing tissues in OCT compound. Slides were cut into 5 µm sections and stained with hematoxylin and eosin and anti-G4 monoclonal antibody for immunohistochemistry. High resolution images of tissues were acquired using an Olympus slide scanner (Bx61VS Olympus America, Center Valley, PA).

### Statistical Analysis

Unless otherwise stated, *in vitro* experiments were conducted in triplicate. Animal experiments were conducted with the *n* as reported in their respective sections (6 unique subjects per group for the subcutaneous model) and (10 unique subjects per group for the orthotopic model). Animal experiments were conducted once in accordance with IACUC approved protocols. Unpaired t-tests and two-way ANOVAs with Tukey’s post-hoc tests were used where appropriate for statistical significance calculations. GraphPad Prism 9.0–10.0 software was used for all statistical analyses. A p-value of less than 0.05 was considered statistically significant).

## Results

### Characterization of RGD-ELNP/miR-200c

**Fig. 1:**
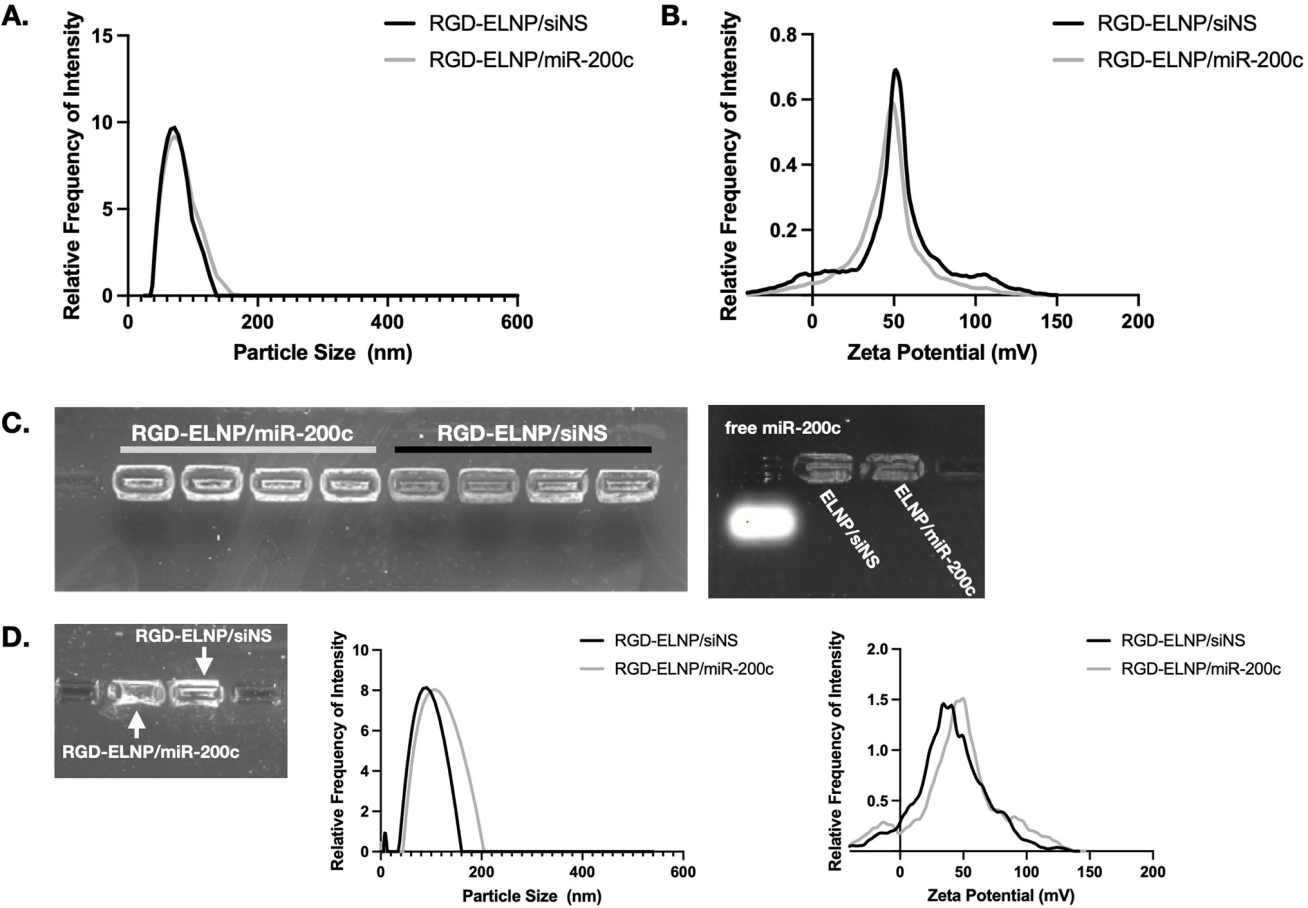
Characterization of the targeted ELNP. (**A**) Size measurements of the targeted ELNPs by hydrodynamic diameter (nm) based on relative frequency of intensity. (**B**) Zeta potential (mV) of RGD-ELNP/miR-200c and siNS nanoparticles. (**C**) Gel electrophoresis shows complete encapsulation of miR-200c or siNS in the targeted ELNP and ELNP. (**D**) Gel electrophoresis, size distribution and charge of RGD-ELNP/miR-200c and RGD-ELNP/siNS at 2 weeks after storage at -80°C.

RGD-ELNP/miR-200c and RGD-ELNP/siNS nanoparticles were reproducibly prepared with an average diameter of 126.1 ± 47.5 nm and 145.4 ± 56.6 nm, respectively (Fig. 1A). Zeta-potential was 25.6 ± 5.9 mV for RGD-ELNP/miR-200c and 15.8 ± 5.9 mV for RGD-ELNP/siNS (Fig. 1B). Agarose gel electrophoresis assays showed efficient RNA encapsulation for ELNP (Fig. 1C). The nanoparticles also had negligible free RNA fallout indicating high RNA loading efficiency and sufficient encapsulation. The targeted ELNP formulations had slight increases in size at -80°C with 5% sucrose as an excipient over the course of 2 weeks, however this was not statistically significant (Fig. 1D).

### Gene Regulation by miR-200c in PDAC Cells

**Fig. 2:**
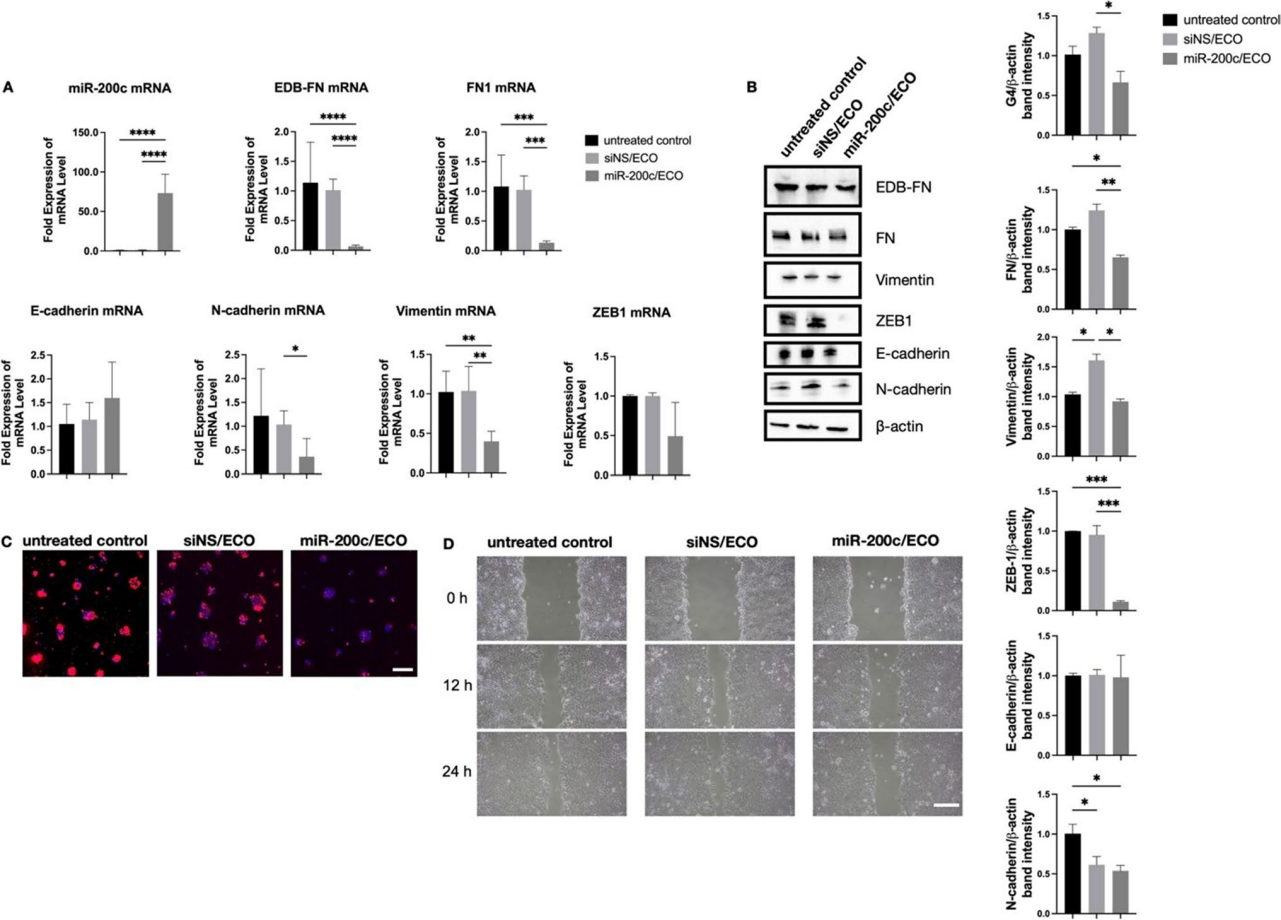
*In vitro* assessment of ECO/miR-200c therapy, ECO/siNS non-specific control, and untreated fresh media control and their effect on FN1, EDB-FN, and the relevant EMT markers and targets. (**A**) qRT-PCR analysis of EDB-FN and EMT markers mRNA expression in KPC-K8484 cells, expression normalized with housekeeping gene GAPDH. (**B**) Western blot images of EMT marker protein levels (EDB-FN, FN, vimentin, E-cadherin, N-cadherin, ZEB1 and β-actin). Densitometry is also shown of proteins, with respect to β-actin bands for each gel (right). (**C**) Confocal fluorescence microscopy of EDB-FN protein expression with ZD2-Cy5.5 staining of 3D spheroids of untreated, siNS/ECO and miR-200c/ECO treated KPC cells. Blue represents nuclei (Hoechst 33342) and red represents EDB-FN (ZD2-Cy5.5). Scale bar is 50 μm. (**D**) Scratch wounds of confluent KPC cells over time in untreated, siNS/ECO and miR-200c/ECO treated media. Scale bar is 100 μm. (Significance is denoted by * *p* < 0.05, ** *p* < 0.01, *** *p* < 0.001, **** *p* < 0.0001).

To determine the effects of miR-200c on gene and protein regulation, well characterized PDAC cells, KPC K8484, were used for *in vitro* experiments. KPC cells were transfected *in vitro* with ELNP/miR-200c to determine gene and protein regulation. ELNP/miR-200c resulted in a significant increase (820%) of intracellular miR-200c compared to the control ELNP/siNS (Fig. 2A). There was significant downregulation of direct miR-200c targets, FN (84.9%), EDB-FN (89%), ZEB1 (50.8%), after treatment with ELNP/miR-200c compared to controls, (Fig. 2A). Similarly, downstream mesenchymal markers, N-cadherin (64.0%) and vimentin (60.0%), also were significantly downregulated, (Fig. 2A). Conversely, the epithelial marker E-cadherin was slightly upregulated with ELNP/miR-200c compared to controls, however not significant (Fig. 2A). At the protein level, EDB-FN, FN, vimentin, ZEB1 and N-cadherin showed a decrease between controls *versus* miR-200c treatment (Fig. 2B). However, E-cadherin remained unchanged, which notably is in alignment with new literature on the role of E-cadherin in cancer [41, 42]. When grown in 3D culture, KPC cells transfected with ELNP/siNS demonstrated stronger ZD2-Cy5.5 staining compared to ELNP/miR-200c, in agreement with the EDB-FN mRNA and protein expression analyses (Fig. 2C). Moreover, treatment with ELNP/miR-200c resulted in reduced migration of the cells over a 24 h period, while siNS had no measurable effect compared to the untreated control (Fig. 2D).

### Therapeutic Efficacy of RGD-ELNP/miR-200c in Mice Bearing Subcutaneous KPC Allografts

**Fig. 3:**
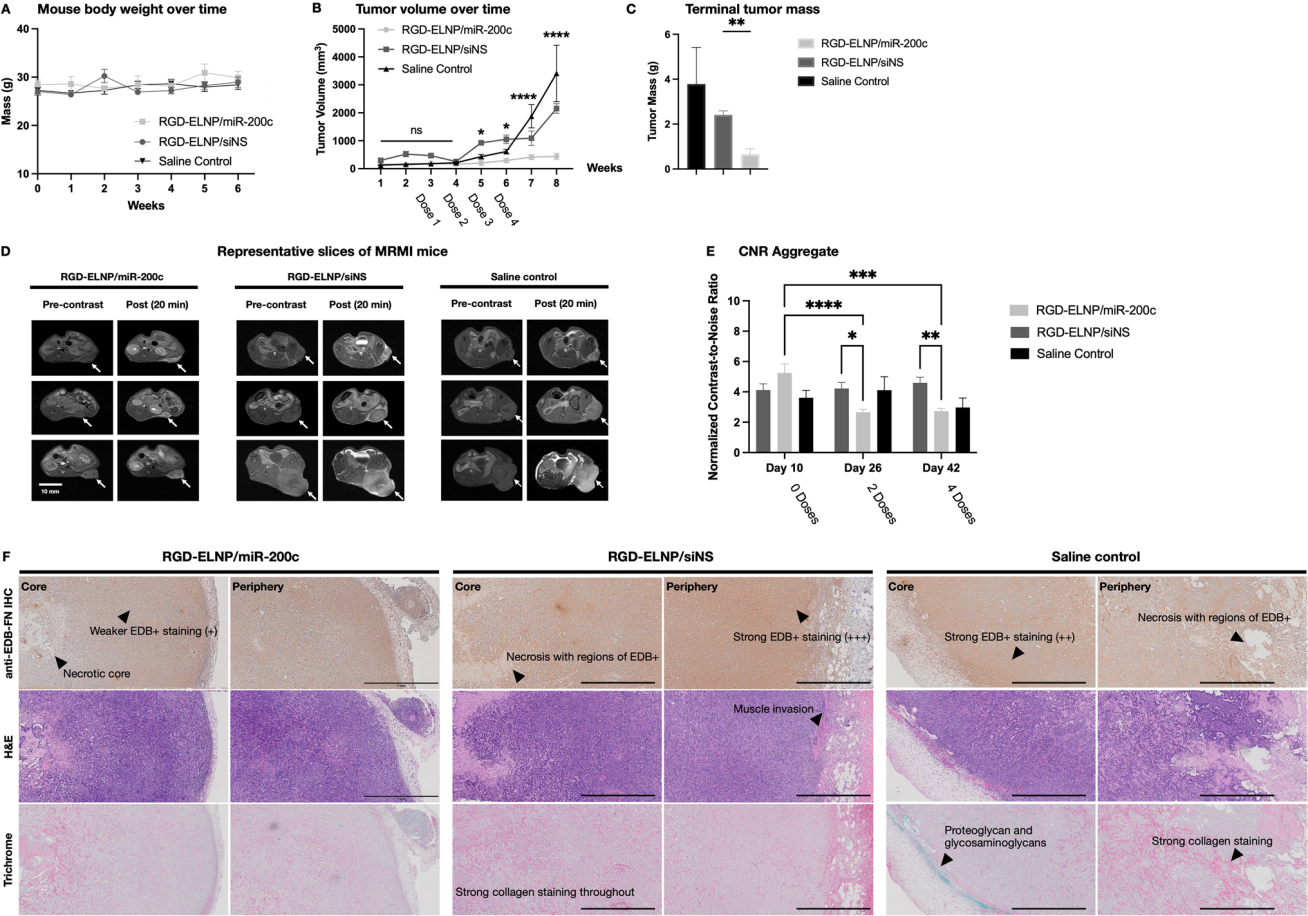
*In vivo* therapeutic monitoring of RGD-ELNP/siNS, RGD-ELNP/miR-200c and saline control in PDAC subcutaneous allografts. (**A**) Mouse body weight for RGD-ELNP/miR-200c and RGD-ELNP/siNS throughout the study (total 6 weeks). (**B**) Caliper measurements of tumor growth over time. (**C**) Differences in tumor masses (g) from tumors extracted when mice reached their experimental endpoints (**D**) MRMI of RGD-ELNP/siNS and RGD-ELNP/miR-200c prior to treatment (day 10), during treatment (day 26) and after treatment (day 42). Scale bar is 10 mm. (**E**) Semi-quantitative CNR analysis of KPC tumors from MRMI with MT218. Graphs show individual multislice CNR from the representative mouse shown (left) and aggregate of CNR for all slices of all tumors from all the mice in the study (right). (**F**) *Ex vivo* histopathology of RGD-ELNP/miR-200c treated tumors (left), RGD-ELNP/siNS treated tumors (middle) and saline control (right). Images are representative sections of tumor stained for H&E (for morphology), IHC with anti-G4 mAb (for EDB-FN) and trichrome RGB (for collagen, non-collagenous protein, and glycoproteins). Histopathology is matched to representative mouse shown in MRMI. Arrows in indicate areas of necrosis and limited tumor invasion in miR-200c treated mice, whereas stronger staining for EDB-FN and fibrosis are apparent in both RGD-ELNP/siNS and saline control. Invasion into muscle was also apparent in both siNS and saline control dosed mice and is marked in the periphery. Scale bar is 1 mm. (Significance is denoted by * *p* < 0.05, ** *p* < 0.01, *** *p* < 0.001, **** *p* < 0.0001).

Next, we assessed the therapeutic efficacy of RGD-ELNP/miR-200c in immunocompetent mice bearing subcutaneous pancreatic tumors using MT218-MRMI. The treatment of the mice with RGD-ELNP/miR-200c or RGD-ELNP/siNS with a total of 4 doses did not show any significant changes in body weight or other overt signs of toxicity, Fig. 3A. The treatment with miR-200c significantly inhibited tumor growth as compared the mice treated with the non-specific RNA (Fig. 3A). Differences in tumor growth between two treatment groups became apparent as early as 2 weeks after the first dose. However, sustained effects from miR-200c resulted in a reduction of tumor growth of 73.0% at week 4 and as much as a 79.6% at week 6 compared to siNS. Excision of tumors at week 6 (day 42 post MRMI) also showed significantly smaller tumors with miR-200c treatment, where miR-200c treated tumors had an average mass of 0.65 g compared to 2.41 g for siNS (Fig. 3B**)**.

Since EDB-FN is a direct target of miR-200c, the effects of miR-200c therapy on the tumors can be determined using MRMI with contrast specific to EDB-FN, namely MT218. In pre-treatment scans of mice, mice slated to enter the miR-200c group showed strong bright signal enhancement throughout the tumors, indicating elevated expression of EDB-FN, over mice assigned to the saline group Fig. 3C. Substantial signal reduction was observed in the miR-200c treated tumors on day 26 and 46 compared to the pre-treated tumors, indicating a reduction of tumor EDB-FN expression with miR-200c treatment, Fig. 3C. The MRMI signal remained unchanged in the siNS treated tumors over the course of dosing and monitoring, similar to the saline control, Fig. 3C.

Semiquantitative analysis of signals through contrast analysis, contrast-to-noise ratio (CNR), showed reduced tumor enhancement in the miR-200c treated group at day 26 after 4 doses (Fig. 3D). At day 26, miR-200c treated mice exhibited a 3.5 CNR-fold enhancement over pre-contrast tumors. Comparatively, siNS treated group had a 5.3-fold normalized CNR. Similarly, miR-200c therapy demonstrated reduced tumor CNR at the week 4 imaging timepoint, although the result did not reach significance. At day 42, miR-200c treated tumors had an average CNR of 2.73 compared to 4.59-fold in tumors treated with siNS nanoparticles. This significant difference in CNR may be due to a reduction in EDB-FN expression from miR-200c treatment. Additionally, less enhancement could be due to compounded by changes in tumor sizes between the siNS group and the miR-200c group, where the miR-200c treated group has substantially smaller tumors than those treated with siNS and the saline controls (Fig. 3A-B).

To confirm our hypothesis about ECM remodeling following a reduction in EDB-FN from miR-200c therapy, we conducted histopathological analysis on tumors obtained on day 42 after the last imaging timepoint. From immunohistopathology of EDB-FN, tumors from all three groups stained throughout for EDB-FN, but miR-200c tumors showed distinctly weaker staining ( +) compared to siNS and saline controls tumors (+ + / +  + +) (Fig. 3E). While siNS and saline controls had higher staining for EDB-FN throughout their tumors, there was not a significant difference between the two. Hematoxylin and eosin (H&E) staining indicated differences in morphological features in the tumors across groups. While all groups presented with distinct adenocarcinoma and were poorly differentiated in H&E staining, miR-200c tumors appeared to have a less dense microenvironment (Fig. 3E). Both control groups had tumors with more stromal regions and areas of necrosis. And in the majority of control tumors some level of muscle invasion into the flank was present. In the miR-200c tumors, the pattern of collagen staining from Sirius red stains may be indicative of tumor remodeling, especially given the weaker staining pattern and the prevalence of gland like structures across approximately half of the tumors.

### Therapeutic Efficacy of RGD-ELNP/miR-200c in Mice Bearing Orthotopic KPC Allografts

**Fig. 4:**
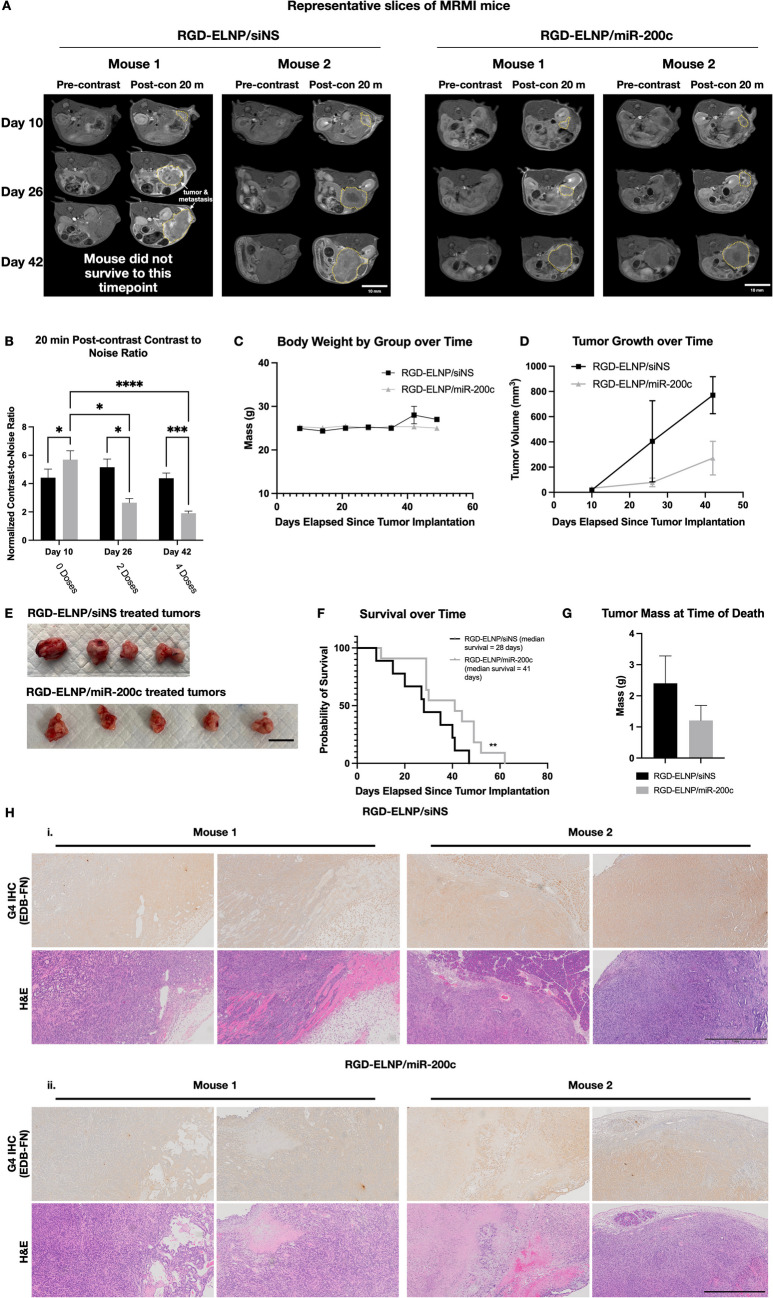
*In vivo* therapeutic monitoring of RGD-ELNP/siNS and miR-200c in PDAC orthotopic (intrapancreatic) allografts. (**A**) MRMI of two mice treated with RGD-ELNP/siNS (left) and of two mice treated RGD-ELNP/miR-200c (right) at three timepoints (before treatment (day 10), during treatment (day 26) and after treatment (day 42)). The majority of RGD-ELNP/siNS mice did not survive till day 42, a representative mouse with metastatic PDAC is shown (mouse 1). Scale bar is 10 mm. (**B**) Graphs show aggregate CNR data from multi-slice analysis of scans of all animal tumors in the study and classified by their respective treatment groups. (**C**) Weekly monitoring of mouse body weight during the experimental study **C.** Tumor volume measurements of tumor growth over time from segmentation of axial FSE MRMI. (**D**) Mouse survival of saline, RGD-ELNP/siNS and RGD-ELNP/miR-200c mice. (**E**) Images of tumors post extraction to demonstrate size differences between tumors of RGD-ELNP/siNS *vs* RGD-ELNP/miR-200c. Scale bar = 1 cm. (**F**) Semi-quantitative CNR analysis of KPC tumors from MRMI with MT218. (**G)** The average mass of extracted tumors at their experimental endpoint. (**H**) *Ex vivo* histopathology of RGD-ELNP/siNS treated tumors (**i**) *versus* RGD-ELNP/miR-200c (**ii**) treated tumors. Images are representative sections of tumor stained for H&E (for morphology) and IHC with anti-EDB-FN mAb (G4). Scale bar is 1 mm. (Significance is denoted by * *p* < 0.05, ** *p* < 0.01, *** *p* < 0.001, **** *p* < 0.0001).

To gain a more physiologically relevant understanding of the effects of miR-200c treatment, we investigated the efficacy of RGD-ELNP/miR-200c in mice bearing orthotopic KPC tumors. MT218-MRMI showed bright signal enhancement in tumors and detected tumor formation in the pancreas on day 10 after tumor initiation. Mice were then treated with RGD-ELNP/miR-200c or RGD-ELNP/siNS by weekly i.v. injection at a dose of 1.0 mg-RNA/kg. MT218-MRMI was performed every 2 weeks after treatment initiation at days 26 and 42. As shown in Fig. 4A, RGD-ELNP/miR-200c resulted in prolonged survival and inhibition of tumor proliferation compared to RGD-ELNP/siNS. MT218-MRMI also revealed that miR-200c treatment resulted in substantial reduction in MR signal brightness in the tumors, while bright signal enhancement remained in the tumors treated with siNS, Fig. 4A. Semiquantitative analysis of tumor MR signal enhancement showed significant CNR decrease in the tumors treated with miR-200c as compared to that with siNS, Fig. 4B. Pre-treatment tumors in the miR-200c group had an average 5.69-fold enhancement over pre-contrast tumors at day 10, which was not significantly different from the siNS (4.41) groups. Post-treatment tumors in the miR-200c treated group only had 1.91-fold CNR increase, which was significantly lower than 4.38-fold CNR increase in the siNS-treated tumors. In addition, more heterogenous signal enhancement patterns were observed in the miR-200c treated tumor than those treated with siNS at day 42. Given the mechanism of action of miR-200c, the reduction of MRMI signal in miR-200c treated tumors is indicative of EDB-FN downregulation mediated by systemic treatment with RGD-ELNP/miR-200c. The heterogeneous enhancement patterns are suggestive of uneven expression of EDB-FN in the tumors.

Therapeutic outcomes were also measured during treatment via body weight (Fig. 4C) and tumor growth (Fig. 4D), and then postmortem through visual assessment of tumors (Fig. 4E), survival (Fig. 4F) and terminal tumor mass (Fig. 4G). Body weight between groups remained consistent over the course of therapy, indicating that administration of nanoparticles did not result in systemic toxicity or promote cachexia, Fig. 4C. Tumor size was estimated from MRMI images at day 10, 26 and 42 Fig. 4D. Growth was attenuated in the miR-200c group, and differences in size were apparent from day 26 measurements. Tumors from a subset of mice that survived to day 42 showed differences in tumor size even when treatment groups are matched for survival, further validating the growth assessment from MRMI that miR-200c delivery results in smaller tumors, Fig. 4E. RGD-ELNP/miR-200c treated mice also had longer survival of a median survival of 41 days than RGD-ELNP/siNS treated mice, which had a median survival of 28 (Fig. 4F). Lastly, the average mass of siNS treated tumors (2.40 g) was double that of miR-200c treated tumors (1.20 g), however this was not significant, Fig. 4G.

The histopathological examination of both saline and ELNP/siNS tumor specimens revealed distinctive features characteristic of adenocarcinoma. The tumors from these two groups displayed a pattern of solid nodules with evidence of general necrosis, suggesting areas of compromised cell viability. The stroma near the necrotic regions exhibited attempts to form glands, albeit with a hollow appearance, indicating the presence of malignant glands. Throughout the tumors, there was a consistent representation of similar tumor cells, with a notable absence of significant stromal elements. Moreover, the histological assessment indicated musculoskeletal invasion, highlighted by the presence of invasive tumor structures and neoplastic vessels. Capillaries were also observed in association with the tumor, and there were indications of invasion into soft tissues, possibly extending into bone or neoplastic bone. The tumor-associated stroma appeared thick, potentially leading to the splitting of skeletal muscle. Overall, these histopathological findings provide a comprehensive view of the tumor architecture, highlighting key characteristics such as glandular formation, invasion into surrounding tissues, and the associated stromal changes associated with PDAC progression *in vivo,* Fig. 4F. Comparatively, miR-200c tumors had substantially more well differentiated glandular structures and, despite also having areas of adenocarcinoma with general necrosis tumors treated with miR-200c, had less invasion into neighboring tissues and displayed less metastases than siNS and saline controls, Fig. 4F.

**Fig 5.**
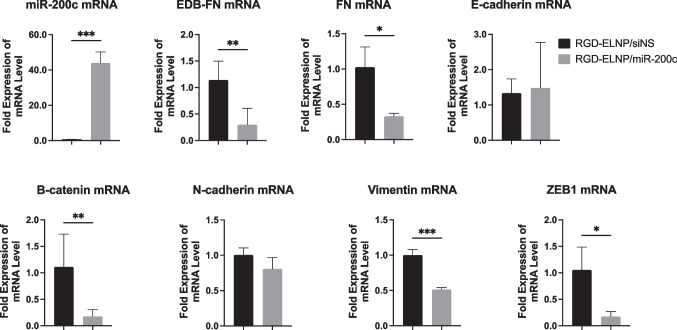
mRNA expression from KPC allograft intrapancreatic tumors at day 42. Tumors were measured for expression of miR-200c and EMT markers (EDB-FN, FN1, E-cadherin, N-cadherin, vimentin and ZEB1). Significance is denoted by * *p* < 0.05, ** *p* < 0.01, *** *p* < 0.001, **** *p* < 0.0001, all PCRs were conducted in triplicate.

Finally, we analyzed the levels of miR-200c and mRNA of some of its downstream gene targets using qRT-PCR. As shown in Fig. 5, qRT-PCR analysis revealed a significant increase in miR-200c in the tumors treated with RGD-ELNP/miR-200c compared to those treated with siNS, suggesting efficient delivery of the therapeutic miRNA via systemic delivery. Treatment with miR-200c resulted in a significant decrease in the expression of EDB-FN and total FN1, as well as other direct and downstream gene targets including β-catenin, ZEB1, and vimentin. However, no significant differences were noted in the expression of E-cadherin or N-cadherin. The decreased expression of EDN-FN corroborated the reduction of EDB-FN observed by MRMI and IHC. The decreased expression of B-catenin, a key player in Wnt signaling, highlighted the regulatory influence of miR-200c. The reductions in ZEB1 expression with miR-200c treatment are indicative of the miRNA's role in suppressing epithelial-mesenchymal transition (EMT). The results suggest that the anti-tumor function of miR-200c may be associated the effective downregulation the down-stream oncogenes by miR-200c. We also observed no change in the body weight of the treated mice, indicating the excellent safety of systemic delivery of miR-200c with RGD-ELNP/miR-200c.

## Discussion

PDAC is a highly desmoplastic cancer, with the dense extracellular matrix (ECM) largely contributing to this fibrotic environment. Desmoplasia has been attributed to a combination of cancer associated fibroblasts, immune cells and ECM proteins, collectively they cause a biologically aggressive, treatment resistant environment which ultimately results in poorer outcomes for patients with PDAC [16, 43]. Hence, PDAC is notoriously difficult to treat with conventional therapies. Given these challenges, remodeling the TME could provide positive outcomes for PDAC. miR-200c is known as a molecular switch for its ability to regulate cellular plasticity through the reversal of epithelial-to-mesenchymal transition (EMT) [44, 45]. However, thus far, there have been a limited number of studies explicitly investigating miR-200c based RNAi delivery for the treatment of pancreatic cancers.

In a previous study, we demonstrated that systemic delivery of miR-200c with targeted RGD-PEG-ECO/miR-200c significantly elevated miR-200c levels in TNBC models in athymic nude mice, resulting in significant downregulation of its downstream gene targets and effective inhibition of tumor proliferation [23]. Using MRMI with a targeted contrast agent, MT218, we showed that miR-200c upregulation resulted in the remodeling of breast cancer TME and significant downregulation of EDB-FN in tumor ECM. In this study, we have investigated the effectiveness of RGD-ELNP/miR-200c on subcutaneous and orthotopic KPC tumors in immunocompetent female and male mice non-invasively with MT218-MRMI. miR-200c is known to be downregulated in PDAC, which contributes to invasion, metastasis, and drug resistance [26]. One of its downstream targets, fibronectin, is overexpressed in PDAC and contributes to fibrotic TME, which supports the invasion and drug resistance of the disease [46–48]. We have shown that systemic treatment of PDAC with RGD-ELNP/miR-200c significantly elevated the level of miR-200c in PDAC tumors and modulated tumor ECM by downregulating EDB-FN expression, resulting in tumor growth inhibition and extended survival.

To establish clinical relevance we utilized the KPC mouse model (Kras^G12D/+^; P53^R172H/+^; Pdx1-Cre), a well-established preclinical model of PDAC that shares key point mutations found in human PDAC, namely Kras^G12D^ and p53^R172H^ [49]. KPC tumors also have the same complex genomic and immune-histological features of human PDAC that result in local immunosuppression and heterogeneity, which allowed for a more accurate and comprehensive assessment of miR-200c in PDAC tumors. Subcutaneous and orthotopic models were used to test the efficacy of RGD-ELNP/miR-200c and determine differences in treatment outcomes between primary or metastatic model tumors. Systemic treatment with RGD-ELNP/miR-200c clearly demonstrated reduced tumor growth, tumor volume, and tumor mass in both. While the difference in tumor size in the intrapancreatic tumors were not statistically significant between treatment groups, the miR-200c treatment did translate to significantly improved survival. Recent studies have established a paradigm that tumor size and overall survival do not correlate, due to the presence of micrometastases but that the fibrogenic subtype can be a key prognosis factor in PDAC survival [6, 50–52]. Further efforts are required to fully investigate the role of fibronectin and the EDB fragment in PDAC survival.

To gain a more comprehensive understanding of the EMT processes and remodeling in the tumor we conducted measured mRNA levels of several key EMT markers. miR-200c delivery also resulted in significant down-regulation of β-catenin, ZEB1, vimentin, and N-cadherin expression in the tumor. β-Catenin is a key player in Wnt signaling, which is associated with tumor carcinogenesis in pancreas and drug resistance of pancreatic cancer [53, 54]. ZEB1, vimentin, and N-cadherin are EMT markers, and their expression is associated with tumor invasive, proliferation, and drug resistance. These findings collectively underscore the efficacy of miR-200c-mediated RNAi in modulating key genes associated with tumorigenesis, potentially inhibiting metastatic processes, and promoting a less aggressive tumor phenotype. Further, downregulation of these direct and downstream targets by elevated miR-200c in PDAC tumors might lead to significant inhibition of proliferation of PDAC. Interestingly, RGD-ELNP/miR-200c resulted in more significant tumor inhibition in subcutaneous models than in orthotopic models, even though significant downregulation of EDB-FN was observed in both tumor models. The results suggest that different environment, including progression through pancreatic ducts, access to vasculature, and various intraabdominal mechanical forces and tissues around the tumors, may also play a role in tumor response to the therapy. Nevertheless, the increase miR-200c and subsequent downregulation of its targets confirmed that RGD-ELNP/miR-200c is effective to deliver therapeutic miRNA in solid tumors across various cancer types via systemic administration [55, 56]. Thus, RGD-ELNP/miR-200c alone or in combination with other therapies is promising to effectively treat PDAC.

MT218-MRMI is a novel non-invasive imaging modality that provides high-resolution, three-dimensional images of EDB-FN in solid tumors. In this study, MT218-MRMI revealed tumor progression, heterogeneity, and specific expression of EDB-FN. Both KPC models exhibited high levels of EDB-FN, consistent with previous findings [57]. EDB-FN, an oncofetal subtype of FN, is uniquely expressed in tumors, correlating with aggressiveness, and absent in normal tissues. RNA-seq analysis and other studies have confirmed its correlation with total fibronectin expression in tumors [34, 57–62]. Thus, high EDB-FN expression detected by MT218-MRMI reflects high total fibronectin expression in tumor ECM. EDB-FN serves as a tumor-specific marker for characterizing tumor ECM and evaluating ECM remodeling in response to therapies. Longitudinal monitoring of tumor response with MT218-MRMI demonstrated a significant reduction in EDB-FN following treatment with RGD-ELNP/miR-200c, indicating tumor ECM remodeling and reduced total fibronectin expression, consistent with mRNA level decreases in extracted tumors. These results were also consistent with our previous observations in TNBC models [23].

Our study further bolsters the utility of MRMI in assessing therapy-induced changes in EDB-FN expression across different cancer types. Because elevated expression of EDB-FN is associated with cancer EMT, angiogenesis, and invasion, MT218-MRMI shows promise for detection and characterization of cancer as well as non-invasive assessment of tumor response to RNA based therapies, including RGD-ELNP/miR-200c, across various cancer types [28, 63]. To verify the effectiveness of MT218-MRMI for assessing PDAC response to RGD-ELNP/miR-200c was in alignment with the results of IHC and H&E staining of tumors in this study, thus, allowing us to validate MRMI with a ground truth. The observed alterations in the TME following miR-200c treatment, including ECM remodeling and changes in cellular density, raise the prospect of clinical translation of MRMI and further serve as another use case for image guidance during therapy. Future clinical studies are warranted to evaluate the long-term safety and efficacy of this treatment and imaging approach.

Our earlier work highlighted the impact of miR-200c on the TME of TNBC, demonstrating alterations in EDB-FN levels and consequent ECM remodeling. This current study extends these findings to PDAC, indicating that delivery of miR-200c can indeed alter the TME by regulating EDB-FN and impacting tumor ECM composition for better therapeutic outcomes. While we focus here on miR-200c’s impact on a subset of EMT associated markers, the role of miR-200c on various components of the TME, including collagens, laminins, and immune circuitry, is under investigated and opens more avenues for research. Understanding the intricacies of these interactions and their modulation by miR-200c expression could provide valuable insights into the complex dynamics of the tumor microenvironment. Moreover, future studies should explore the long-term effects of miR-200c therapy, potential resistance mechanisms, and the interplay with other therapeutic modalities. Investigating the broader implications of miR-200c expression on the tumor-immune microenvironment crosstalk is crucial for developing comprehensive therapeutic strategies.

## Conclusion

The findings presented in this study, along with our prior research, hold significant implications for advancing cancer therapeutics and imaging. The success of RGD-ELNP/miR-200c nanoparticles in downregulating EDB-FN and suppressing tumor growth, demonstrated in both subcutaneous and orthotopic PDAC models and previously in TNBC, underscores the versatility and potential applicability of this therapeutic strategy across different cancer types. Moreover, miR-200c therapy has shown efficacy in modulating PDAC tumors within an immunocompetent environment. However, further investigation is needed to elucidate the mechanisms affecting PDAC and miR-200c in subcutaneous *versus* orthotopic tumors. Despite this, miR-200c presents an opportunity for combination therapy to enhance the efficacy of other treatments or leverage stromal remodeling, broadening the scope of miR-200c-based therapies and suggesting its potential effectiveness in a diverse set of malignancies.

The integration of MRMI with MT218 for non-invasive assessment of tumor response to miR-200c therapy is a notable advancement. This imaging technique, previously applied to TNBC and now successfully extended to PDAC, serves as a crucial tool for monitoring therapy-induced changes in EDB-FN expression. Its utility in providing 3D high-resolution images enhances our ability to understand treatment outcomes non-invasively and in real-time. EDB-FN’s impact on the TME, can be corroborated across different cancer types, and further can be validated in immunocompetent models where the TME is much more complex. The nuanced understanding of miR-200c's role in ECM remodeling and its potential influence on various components of the TME adds depth to the literature, providing a foundation for future investigations in cancer biology and therapeutic development.

In conclusion, these findings represent a step towards efficacious and novel therapies for PDAC and establish that miR-200c delivery can modulate the TME. The successful application of advanced imaging techniques further emphasizes the importance of non-invasive tools to unraveling the complexities of tumor responses, guide future therapeutic endeavors, and ultimately impacting clinical practices in oncology.

## Supplementary Information

Below is the link to the electronic supplementary material.

Supplementary file1 (XLSX 10.1 KB)

## Data Availability

The authors declare that the data that supports the findings of this study are included within this paper and its Supplementary Information files.
